# Surgical Anatomy of Vertebral Artery in Relation to Atlantoaxial Instrumentation: A Cadaveric Study

**DOI:** 10.7759/cureus.35949

**Published:** 2023-03-09

**Authors:** Mukesh Singla, Pankaj Kandwal, Rashmi Malhotra, Mohd S Ansari, Rajnish K Arora, Kanchan Bisht, Brijendra Singh

**Affiliations:** 1 Anatomy, All India Institute of Medical Sciences, Rishikesh, IND; 2 Orthopaedics, All India Institute of Medical Sciences, Rishikesh, IND; 3 Anatomy, Jawaharlal Nehru Medical College, Aligarh Muslim University, Aligarh, IND; 4 Neurosurgery, All India Institute of Medical Sciences, Rishikesh, IND

**Keywords:** atlanto-axial instrumentation, atlas, axis, vertebral artery injury, surgical anatomy

## Abstract

Background: With the advent of pedicle screws and advanced instrumentation techniques, internal fixation and stabilization of upper cervical vertebrae are possible in fractures of an axis. However, the proximity of vertebral arteries (VAs) poses a unique challenge to surgeons during these procedures and can result in profound physical impairment to patients. Cadaveric studies contributing to fine anatomical details necessitate conducting such studies.

Methods: After receiving due ethical permission, this descriptive cross-sectional study was carried out on 10 cadavers in the department of Anatomy, All India Institute of Medical Science (AIIMS) Rishikesh. Twenty VAs were dissected along their course, and measurements of parameters related to the axis and atlas vertebra were noted.

Results: The length of the pre-osseous segment related to the axis (VAX-1) on the right and left sides were from 3.8 to 14.5 mm (7.48±3.88 mm) and 4.46 to 10.5mm (6.94±2.01mm) respectively. The length of the osseous segment related to the axis (VAX-2) on the right side and left sides were from 6.82 to 31 mm (17.9±7.84mm) and 7.35 to 20 mm (15.6±4.53). The osseous segment of the VA related to the axis (VAX-2) shows genu (bend), which extends to a variable distance towards the midline. The mean distance of VA genu from the midline of the axis vertebral body on the right and left sides was 15.6mm and 17.5 mm, respectively. The percentage of superior articular facet (SAF) surface area of the axis occupied by the VA was 25-50% in nine and 50-75% in 11 cadavers, reflecting incomplete occupancy.

Conclusion: The study suggests that for instrumentation of the axis vertebra in the midline, the minimum distance between the genu of both sides of VA segments, related to an osseous segment of the axis (VAX-2) and medial extent of the VA groove of the atlas, should be considered as a safe zone to minimize inadvertent VA injury. During atlantoaxial fixation through a posterior approach in interarticular, pars, and pedicle screws, the surgical anatomy of the VA in relation to the osseous segment of the VA within the transverse process of the axis should be kept in mind to avoid inadvertent VA injury.

## Introduction

Surgical anatomy

The vertebral artery (VA) is a branch of the first part of the subclavian artery and travels through the transverse foramen in the cervical spine (C6-C1). It terminates by joining the opposite VA to form the basilar artery in the cranial cavity (1). The VA has been divided into four segments by earlier authors [1-4]; traditionally, surgeons follow the given subdivision mentioned as V1-origin to entry in C6 vertebra; V2-C6 transverse foramen to C1 transverse foramen; V3-C1 foramen to entry into dura or between the transverse process of C2 and dura mater; and V4-intracranial [2,3].

The first or prevertebral segment of the VA commences from the subclavian artery to C6 cervical vertebra, while the second segment of the artery runs cranially in a vertical manner within the transverse foramen from the C6 vertebrae to C3. The VA exits the C2 foramen by taking a sharp bend medially at the superior articular facet of the axis and enters the transverse foramen of C1. It then runs in a groove present on the superior surface of the posterior arch of the atlas. The fourth segment of the artery crosses the atlantooccipital membrane and enters the cranial cavity [4,5]. As the VA ascends cranially, the transverse foramen moves from an anterolateral to a posteromedial position [5].

The first (atlas) and second (axis) cervical vertebrae are considered atypical cervical vertebrae. Lateral masses replace the vertebral body in the atlas joined together by anterior and posterior arches, and there is a groove for the VA on the superior aspect of the posterior arch. The axis vertebra also shows an atypical feature in having a vertical, tooth-like projection named the odontoid process or dens in the center and two lateral masses [6]. With the increasing trend of cervical spinal instrumentation, such as posterior fixation and pedicle (including pars, pedicle, and transarticular screws) and anterior odontoid screws, the VA is vulnerable to inadvertent iatrogenic injury [7]. Moreover, anomalies of VA segments related to the axis and atlas show an increased incidence of variations. Hence, the course, relations, and variations of the VA in the context of the cervical region demand great attention.

The axis vertebrae could be involved in injuries such as odontoid fractures as well as combined atlas and axis fractures. Earlier studies have reported that fractures of the odontoid process are common, accounting for 10% to 20% of all cervical spine fractures, and that posterior fusion in type II odontoid fractures carries a risk of injury to the VAs and exiting nerve roots [8,9]. A study conducted on 116 upper cervical spine injuries reported that 31 of these injuries had combined C1-C2 fractures. These included 70% of atlas fractures, 30% of odontoid fractures, and 30% of C2 traumatic spondylolisthesis [10].

One study reported that for atlantoaxial instrumentation, transarticular screws enter the lateral mass of the axis, and pass through the isthmus of the axis and the posterior part of the atlantoaxial articulation, finally reaching the lateral mass of the atlas. Course and relation of the VA within the axis are fundamental to avoid any inadvertent injury and ensure the safe placement of screws [11]. The course of the VA follows an oblique manner through the axis. It has been reported that posterior C1-C2 transarticular screw fixation could result in VA injury, which may have life-threatening complications [12]. VA injuries commonly occur because of faulty instrumentation in the VA groove of the axis, as it is related to a groove on the inferior surface of the superior articular facet of the lateral mass of the axis. Screws applied to the axis may cause complications due to their close relationship with the VA and the subsequent changes in their course in relation to the axis [13-16].

Vaccaro et al. suggested that radiological evaluation before surgery through a computed tomography (CT) angiogram can detect anomalies of the V2 segment of VAs preoperatively [17]. However, there are certain limitations to radiological evaluation: the adverse events that may be encountered in a CT angiography of the cervical spine include allergic reaction to contrast, renal failure in patients with deranged renal function, and an increased risk of thyroid cancer owing to the radiation dose. It is an expensive investigation, and this facility is lacking at many healthcare centers [18].

The various techniques for atlantoaxial fixation include posterior clamps, posterior wiring techniques, C1-C2 transarticular screw fixation, posterior C1 lateral mass screw with C2 pars or pedicle screw fixation, and anterior transoral C1 lateral mass to C2 vertebral body fixation [19].

A significant drawback of atlantoaxial transarticular screw fixation is the potential risk of VA injury, especially with a high-riding type [20]. Moreover, C2 pedicle screws or transarticular atlantoaxial screws are technically demanding and carry an increased risk of VA injury [21].

Therefore, it is imperative that surgeons perform the operation within a safe zone, especially for anterior screw fixation in case of Odontoid fractures, posterior transarticular screw fixation, pedicle, and pars screws, so that even if no angiography is conducted, VA injury may be prevented. The present cadaveric study was carried out to provide a database regarding parameters pertinent to atlantoaxial instrumentation and the safety of the VA in such surgeries.

## Materials and methods

This descriptive cross-sectional study was carried out in the Department of Anatomy, All India Institute of Medical Sciences (AIIMS) Rishikesh, after obtaining sanction and approval from the Institutional Research and Ethical Committee, letter no: IEC/IM/111/RC60/2014. A careful dissection of the VA along its course was conducted in ten adult male cadavers. Silicone was injected into the arteries, which were subsequently painted with red color. The surgical anatomy of the VA was evaluated along its course from the C3 transverse process to its entrance into the foramen magnum. The course and relations of the VAs on both sides were noted, with particular emphasis on their relationship with bony landmarks and anatomical parameters of the atlas and axis, as mentioned in the results. All linear measurements were taken for both sides with the help of electronic digital calipers accurate to 0.01 mm. Additionally, a goniometer was used for angular measurements.

Cadaveric dissection

**Figure 1:**
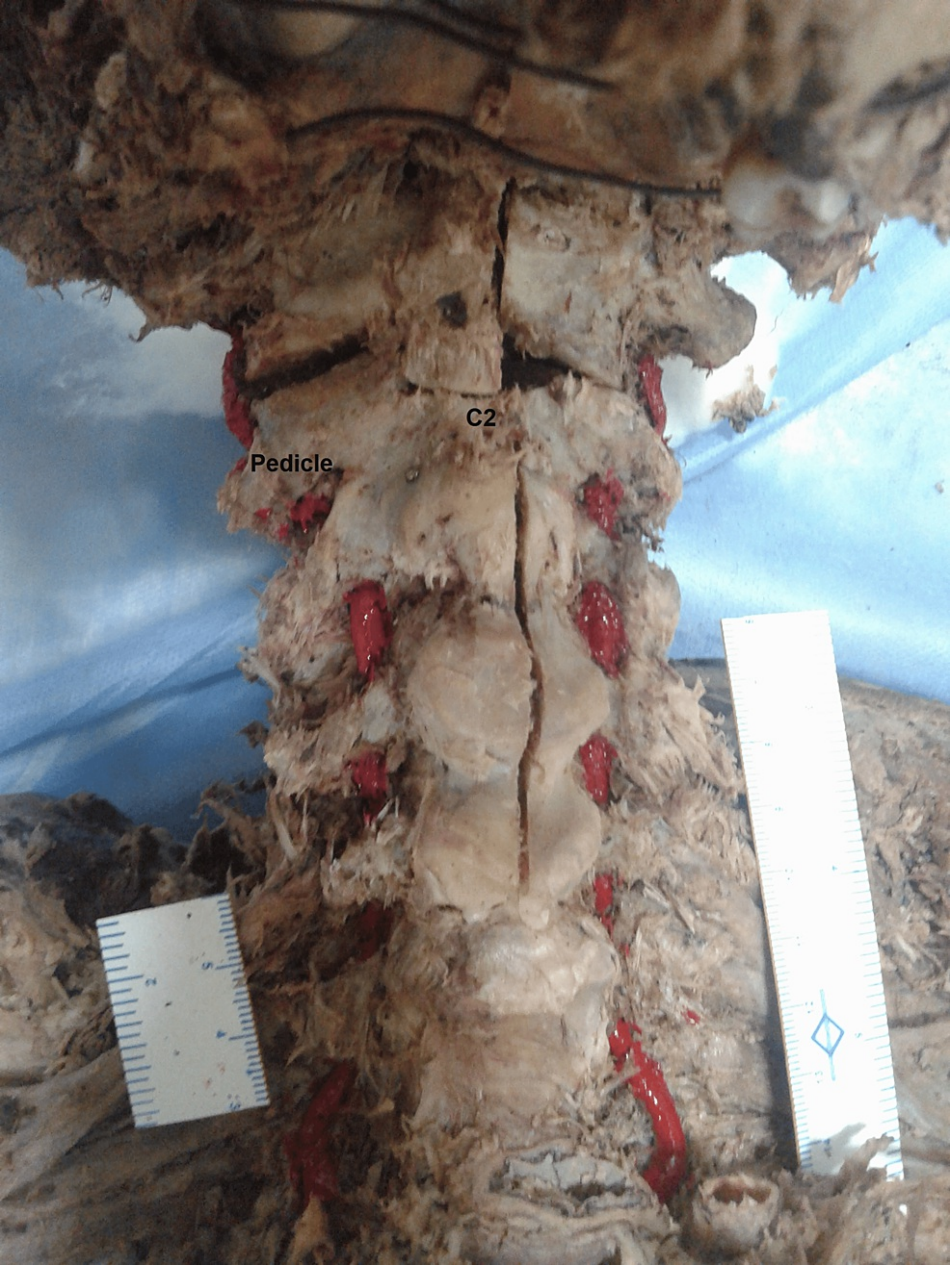
Anterior view of the neck showing vertebral arteries passing through foramina transversaria of C6-C1

After placing the cadaver in the prone position, a midline incision was made from the inion of the occipital bone to the vertebra prominens of C7. Posterior dissection of the neck muscles was carried out and the suboccipital triangle was exposed after dissecting trapezius, splenius capitis, and semispinalis capitis. Anterior dissection of the neck in a posterior triangle was also carried out along the commencement of the VA from the first part of the subclavian artery and traced cranially towards the transverse foramina of the cervical vertebrae. This study focused on the course and relation of the VA after its exit from the transverse foramen C3 and further upward its course. The pre-osseous course of VA from its exit from C3 to its entry into the transverse foramen of C2 was named VAX-1. De-roofing of the pedicle was done, and the osseous course within the C2 transverse process was measured. The length of the osseous segment of the VA was measured from the de-roofed pedicle of C2 till VA entered the C1 transverse foramen; this was named VAX-2. After defining the bony landmarks and VA segments, measurements were recorded and photographs were taken during the stepwise dissection, as depicted in Figure 1.

## Results

VA segment between C3 vertebra and the axis (C2)

The VA traversed through the transverse foramen of C3 to C2 vertebrae bilaterally in all specimens surrounded by a rich venous plexus. The length of the pre-osseous segment VAX-1 on the right side ranged from 3.8 to 14.5 mm (7.48±3.88 mm) and on the left from 4.46 to 10.5 mm (6.94±2.01mm). The osseous segment length VAX-2 ranged from 6.82 to 31 mm (17.9±7.84mm) on the right side and 7.35 to 20 mm (15.6±4.53) on the left. The distance of genu of the VA from the midline of the C2 body (dgen1) on the right side was from 13.5 to 17.39 (15.6±1.22mm) and on the left from 15 to 20.3 mm (17.5±2.07mm). The distance of genu of the VA from the articular surface of the superior articular facet (SAF) of the axis (dgen2) on the right side ranged from 4.8 to 10.1 mm (7.33±2.15) and on the left from 5.2 to 10 mm (7.14±2.31mm). The transverse diameter of the SAF of the axis (tsaf) on the right side ranged from 15.3 to 19.5mm (average 17.7±1.31mm) and on the left from 14.1 to 18.5 mm (17.0±1.63mm). The occupancy of SAF by the VA (occsaf) on the right side ranged from 5 to 10.3 mm (8.89 ±2.25mm) and from 6.5 to 11.5 mm on the left side (10.3± 3.55mm). The percentage of the transverse extent of the VA occupying SAF (pertrocc) on the right side ranged from 28.9 to 59.4% (49.14± 12.3%) and 35.9 to 65.7% (53.43± 10.8%) on the left. The angle of the genu of the osseous part (VAX-2 segment) inside the transverse foramen and on the inferior surface of the superior articular facet of the axis (SAF, anggen) ranged from 65 to 115 degrees (87±17.5 degrees) on the right side and 70 to 110 degrees (91.4±13 degrees) on the left. The mean, median, and standard deviation were calculated for each side's parameters. Paired sample t-test, Student’s t-test, and Wilcoxon W were used to detect significant differences in the obtained values of the VA segments in the compared groups (P=<0.05). Anatomical measurements of different parameters of the VA in relation to the axis and their statistical analysis are shown in Tables 1-2.

**Table 1:** Anatomical measurements of the different parameters of the vertebral artery in relation to the axis vertebra r: right, l: left VAX-1: Preosseous length of the vertebral artery (VA) segment related to the axis VAX-2: Osseous + post-osseous length dgen1: Distance of genu from the midline of the vertebral body dgen2: Distance of genu from the superior articular facet (SAF) tsaf: Transverse diameter of the SAF occsaf: Occupancy of the SAF by VA pertrocc: Percentage of occupancy of the SAF by VA anggen: Angle of genu

VXA-1r	VXA-1l	VAX-2r	VAX-2l	dgen1r	dgen1l	dgen2r	dgen2l	tsafr	tsafl	occsafr	occsafl	pertroccr	pertroccl	anggenr	anggenl
	10	10	10	10	10	10	10	10	10	10	10	10	10	10	10
Mean 7.48	6.94	17.9	15.6	15.6	17.5	7.33	7.14	17.7	17.0	8.89	10.3	0.492	53.4	87.4	91.4
Median 6.21	6.47	17.3	16.4	15.9	17.6	7.10	6.30	18.1	17.8	9.25	10.9	0.523	56.3	85.0	88.0
Standard 3.88	2.01	7.84	4.53	1.22	2.07	2.15	2.31	1.31	1.63	2.25	3.55	0.123	10.8	17.5	13.0
Minimum 3.80	4.46	6.82	7.30	13.5	14.5	4.80	5.20	15.3	14.1	5.00	6.50	0.289	37.0	65	70
Maximum 14.5	10.5	31.0	20.0	17.4	21.0	10.1	11.5	19.5	18.5	11.8	18.5	0.677	65.7	115	110

**Table 2:** Statistical analysis of the distance between the genu of the vertebral artery segment VAX (axis) of the right and left sides

N		10
Mean		14.4700
Median		13.4000
Mode		10.00^a^
Std. Deviation		4.25938
Minimum		10.00
Maximum		21.50
Percentiles	25	10.7500
	50	13.4000
	75	18.5750
a. Multiple modes exist. The smallest value is shown		

VA segment between the axis and atlas (VAt)

The segment of the VA related to the atlas (VAt) initially courses laterally after exiting the transverse process of the axis. The lateral edge of the C2 Ganglia was related to the VAt segment of the VA at a distance of 5 to 14.5 mm (average 9.1mm). Considering the total length of the VA segment between the axis and atlas, the VAt ranged from 7 to 14.8 (average 11.6mm). The VA groove on the superior surface of the posterior arch of the atlas was occupied to an extent of 26.66-70% (average 50.27%) on the right side and 24.5-75% (avg 51.68%) on the left. The lateral edge of the C2 ganglia was related to the VAt segment of the VA at a distance of 5 to 12.8 mm (average 8.83mm) on the right side and 5 to 14.5 mm (average 9.39mm) on the left. The distance of the VA from the lateral end of the dural tube was 9.3 to 17.8 (average 14.23mm) on the right side and 9.4 to 16.5mm (average 13.63mm) on the left. No specimen gave off the posterior inferior cerebellar artery extracranially.

Different parameters of the VA in relation to the atlas and their statistical analysis are shown in Tables 3-4.

**Table 3:** Different parameters of the vertebral artery in relation to the atlas vertebra

Variable	Mean (mm)	Std. Deviation	P value
Total length of the V2-segment b/w C2-C1 (R)	10.910	2.1810	.011
Total length of the V2-segment b/w C2-C1 (L)	12.200	1.8625	
Distance of the lateral edge of the C2 ganglion from the vertebral artery (R)	8.8320	2.75722	.095
Distance of the lateral edge of the C2 ganglion from the vertebral artery (L)	9.3860	3.41105	
Distance of the artery from the lateral end of the dural tube (R)	14.2250	3.08808	.289
Distance of the artery from the lateral end of the dural tube (L)	13.63	2.415	
Percentage (R)	50.271	18.7433	.551
Percentage (L)	51.69	19.329

**Table 4:** Paired t-test (N=10); Statistical analysis of parameters of vertebral artery related to atlas vertebra

Variable	Mean	Std. Deviation	P value
Total length of the V2-segment b/w C2-C1 (R)	10.910	2.1810	.011
Total length of the V2-segment b/w C2-C1 (L)	12.200	1.8625	
Distance of the lateral edge of the C2 ganglion from the vertebral artery (R)	8.8320	2.75722	.095
Distance of the lateral edge of the C2 ganglion from the vertebral artery (L)	9.3860	3.41105	
Distance of artery from the lateral end of the dural tube (R)	14.2250	3.08808	.289
Distance of artery from the lateral end of the dural tube (L)	13.63	2.415	
Percentage (R)	50.271	18.7433	.551
Percentage (L)	51.69	19.329

## Discussion

A precise knowledge of the surgical anatomy of the VA and its relationship to the upper cervical vertebrae is necessary to prevent its inadvertent injury during instrumentation [6]. The findings in a study conducted by Vaccaro AR et al. suggested that the distance between the transverse foramen of the right and left sides decreased from 29 mm at C6 to 26 mm at C3 on average, and the distance between the posterior wall of the vertebral body from that of the transverse foramen decreased from 3.5 mm at C6 to 2.2 mm at C3 [17]. The present study focused on the parameters after VA exits from C3 transverse foramen and in relation to the axis (C2). Taitz and Arensburg studied 36 VAs in 18 cadavers; they observed that 14 VAs had varying degrees of tortuosity, and four had marked kinking at the transverse foramen of the axis and caused concomitant erosion in this part of the bone [15]. In the present study, we did not find any marked kinking or erosion; however, bend or genu was seen at the transverse foramen of the axis.

In a study carried out by Cacciola et al. on 20 VAs, the average length of the VA segment from C3 to C2 vertebrae was 23.4 mm; in the present study, it was 23.9 mm, similar to their values [3]. The lengths of the pre-osseous and osseous segments of the artery related to the axis were 11.3 mm and 14.8 mm [3]. The present study recorded the lengths of the pre-osseous (VAX-1) and osseous (VAX-2) segments as 7.2 mm and 16.7 mm, respectively when all 20 VAs were considered irrespective of sides. In the present study, parameters were measured bilaterally. The length of the pre-osseous segment VAX-1 on the right side ranged from 3.8 to 14.5 mm (7.48±3.88mm) and on the left side from 4.46 to 10.5 mm (6.94±2.01mm). The osseous segment length VAX-2 ranged from 6.82 to 31 mm (17.9±7.84mm) on the right side and 7.35 to 20 mm (15.6±4.53) on the left.

Cacciola et al. reported that the distance of the tip or the most medial part of the VA osseous segment from the midline of the C2 body ranged from 6.1 to 16.2 mm (11.7mm average) from an anterior transoral surgical view. Cruz-Elizondo et al. recorded the distance between C2 spinous process towards the medial edge of the most prominent point of the proximal loop as 43.8±4.2 mm [5]. Güvençer M et al. carried out an angiographic study in 12 human cadavers and reported that the distance between the VA's medial side of the V2 segment related to the axis and the midline was 17.6±6.1 on the right side and 14.7±2.1 on the left. The mean distance from the midline to the most posteromedial edge of the loop was 25.6 ± 3.5 mm (range 20-35 mm) on the left side and 30.4±3.8 mm (range 23-36 mm) on the right [9]. In the present study, the distance of VA bend/genu from the midline of the C2 body on the right side ranged from 13.5 to 17.39 (15.6±1.22mm) and on the left from 15 to 20.3 mm (17.5±2.07mm), which is different from the previously mentioned study in terms of the right and left sides; there was a significant difference on both sides regarding this parameter (Student’s t-test p-value 0.010 and Wilcoxon W 0.019). In an anterior transoral surgical approach for drilling into the body or pars interarticularis, this parameter can play a significant role, as the screws can be placed safely within the medial safe zone, the lateral limit of which can be the medial one-third margin of the SAF of C2.

Cacciola et al. reported the distance of genu of the VA from the articular surface of the SAF of the axis as ranging from 0.6-4.8 (2.5mm average). The authors observed that on the right side, the values ranged from 4.8 to 10.1mm (7.33 ±2.15), and on the left side, from 5.2 to 10 mm (7.14±2.31 mm) [3].

In C6-C3, the VA is related to the transverse process closer to the lamina. In C2, the course of the VA in the SAF of the axis is very close to the vertebral body and not the lamina, making the VA prone to injury with transarticular and inter-articular screw implantation techniques. Cacciola et al. stated that screw placements during interarticular fixation are directed towards the C2 vertebral body and in the SAF of C2 during transarticular fixation. Other studies have also reported susceptibility to VA injury during these procedures [22-24]. In our study, we too found the VAs on both sides in close relation with the inferior aspect of the SAF of C2.

In 2004, Cacciola et al. reported that the transverse extent occupancy of the SAF of C2 by the VA was more than 75% in two, 50-75% in five, 25-50% in eight, and less than 25% in five specimens. In the present study, it was 25-50% in nine and 50-75% in 11 specimens [3]; these values were measured bilaterally, differing from Cacciola et al.’s method. In this study, the transverse diameter of the SAF of the axis on the right side ranged from 15.3 to 19.5mm (17.7±1.31mm) and on the left from 14.1 to 18.5 mm (17.0±1.63 mm) and was significantly different on right and left sides (p-values: (AL3) Student’s t-test <0.001, Wilcoxon W test 0.002). Occupancy of the SAF by the VA on the right side ranged from 5 to 10.3 mm (8.89±2.25mm) and 6.5 to 11.5 mm on the left (10.3±3.55 mm) and the difference between the right and left sides was significantly different in the present study (p-value: Student’s t-test <0.001, Wilcoxon W test 0.002). The percentage of the transverse extent of the VA occupying the SAF on the right side ranged from 28.9 to 59.4% (49.14±12.3%) and 35.9 to 65.7% (53.43±10.8%) on the left side, showing a significant difference in the present study (p-value: Student’s t-test <0.001, Wilcoxon W test 0.002).

Angle of genu

In their study, Cruz-Elizondo et al. measured the angle of the proximal loop of the VA segment related to the axis V3 as 112.60 degrees ±5.66 on the right side and 114.60±6.02 on the left. Cacciola et al. measured the angle of the loop formed by VA within bony foramen on the inferior surface of the SAF to be 2-110 degrees (86 degrees average) [5]. Alfaouri and Hadidi measured the mean angle (a-1) between the ascending and descending bends of the loop formed by the VA segment related to the axis (V3) to be 72.13 degrees ±1.48 on the right and 75.66±1.57 on the left [1]. Güvençer et al. measured the angle between the V2 segment of the VA and the midline as 4.0±1.9° on the right and 2.2±1.4° on the left side [9].

In the present study, the angle of genu was defined as the angle formed when the VA takes a lateral bend after forming the genu (inside the transverse foramen and on the inferior surface of the SAF of the axis in the osseous part (VAX-2 segment); it ranged from 65 to 115 degrees (87.4).

Regarding C1-C2 articulation, the inferior facet of C1 is closely related to the VA segment VAt from the axis to the atlas. The mean length of the VA segment between C1 and C2 was 9.7 mm on the right side and 10.9 mm on the left [25]. In a study conducted by Arthur et al., the mean length of this segment was 9.8 mm on the right side and 11.7 mm on the left. In the present study, the mean length was 11.6 mm on both sides [26].

After emerging from the transverse foramen of C1, the VA takes a medial loop-like course on the superior aspect of the posterior arch of the atlas in a groove. Arthur et al. mentioned that this VA segment is prone to injury during C1-C2 transarticular screws or C-2 pars screws [26]. In our study, the VA formed a loop and occupied variable space in all the specimens on the left and right sides. Cacciola et al. reported 42-71% (avg 57%) occupancy of the groove in their study. In our study, the VA occupancy percentage within the bony groove on the superior aspect of the atlas ranged from 26.66 to 70% (avg 50.27%) [3]. Furthermore, the distance of the most medial edge of the VA groove on the outer cortex of the posterior arch of the atlas from the midline of the posterior tubercle was 17.2 mm and 16.7 mm on the right and left sides, respectively. Khanfour et al. reported their results as 15.0 mm on the right side and 16.0 mm on the left [25].

Gamal H. el-Sayed Hassanein reported on the ways to avoid VA injury in screw placement in the posterior arch of the atlas; the dissection should remain within 10 mm and 6 mm lateral to the midline on the posterior and superior aspects, respectively [27]. In the posterior approaches of atlas surgery, a safe distance to prevent VA injury is 15 to 20 mm lateral to the midline [2]. The safe zone should not exceed 17mm lateral to the midline and should remain 8 mm within the midline while dissecting the superior aspect of the atlas [28].

Fayza A. et al. stated that injury to the VA can be avoided if the surgical lateral exposure of the posterior arch is a maximum of 17.3 mm from the midline and dissection on the superior aspect of the posterior ring of the atlas is well within 7.5 mm from the midline [29].

In our study, the distance of the VA from the lateral end of the dural tube was 14.23 mm on the right and 13.63 mm on the left side. Cacciola et al. reported this distance as 14.7-17.9 mm (avg. 15.3 mm) [3].

The superior cervical sympathetic ganglion is related laterally to the VAt segment of the VA. A previous study has reported this distance to be 1.4 mm (on the right) and 1.7 mm (on the left) [25]. We calculated this distance as 8.83 mm on the right side and 9.39 mm on the left.

## Conclusions

The findings of the present study suggest that the distance between the medial genu of the osseous segment of VA in relation to the axis and the medial extent of the VA groove of the atlas may be considered a safe zone in anterior midline approaches, inside which the placement of screws or instrumentation is least likely to cause an injury to the VA in atlantoaxial instrumentation. During posterior and interarticular approaches with pars and pedicle screws, the surgical anatomy of the VA in relation to the osseous segment of the VA within the transverse process of the axis should be kept in mind to avoid inadvertent VA injury.

## References

[1] Alfaouri-Kornieieva M, Al-Hadidi AM (2014). Morphology of the vertebral artery in Asian population. Asian J Med Sci.

[2] Bruneau M, Cornelius JF, Marneffe V, Triffaux M, George B (2006). Anatomical variations of the V2 segment of the vertebral artery. Neurosurgery.

[3] Cacciola F, Phalke U, Goel A (2004). Vertebral artery in relationship to C1-C2 vertebrae: an anatomical study. Neurol India.

[4] Campero A, Rubino PA, Rhoton AL (2010). Anatomy of the vertebral artery.

[5] Cruz-Elizondo MA, Villarreal-Silva EE, Quiroga-Garza A (2020). Safety ranges in v3 segment of the vertebral artery for surgical procedures at the craniocervical junction. Int J Morphol.

[6] Devin CJ, Kang JD (2009). Vertebral artery injuries in cervical spine surgery. Instr Course Lect.

[7] Formentin C, Andrade EJ, Maeda FL, Ghizoni E, Tedeschi H, Joaquim AF (2019). Axis screws: results and complications of a large case series. Rev Assoc Med Bras (1992).

[8] George B, Cornelius J (2001). Vertebral artery: surgical anatomy. Oper Tech Neurosurg.

[9] Güvençer M, Men S, Naderi S, Kiray A, Tetik S (2006). The V2 segment of the vertebral artery in anterior and anterolateral cervical spinal surgery: a cadaver angiographic study. Clin Neurol Neurosurg.

[10] Lu J, Ebraheim NA (1999). The vertebral artery: surgical anatomy. Orthopedics.

[11] OʼDonnell CM, Child ZA, Nguyen Q, Anderson PA, Lee MJ (2014). Vertebral artery anomalies at the craniovertebral junction in the US population. Spine (Phila Pa 1976).

[12] Puschak TJ, Anderson PA (2002). Posterior C1-C2 transarticular screws. Tech Orthop.

[13] Rao G, Apfelbaum RI (2005). Odontoid screw fixation for fresh and remote fractures. Neurol India.

[14] Schroeder GD, Hsu WK (2013). Vertebral artery injuries in cervical spine surgery. Surg Neurol Int.

[15] Taitz C, Arensburg B (1991). Vertebral artery tortuosity with concomitant erosion of the foramen of the transverse process of the axis. Possible clinical implications. Acta Anat (Basel).

[16] Tenny S, Munakomi S, Varacallo M (2022). Odontoid fractures.

[17] Vaccaro AR, Ring D, Scuderi G, Garfin SR (1994). Vertebral artery location in relation to the vertebral body as determined by two-dimensional computed tomography evaluation. Spine (Phila Pa 1976).

[18] Ryken TC, Hadley MN, Aarabi B (2013). Management of isolated fractures of the axis in adults. Neurosurgery.

[19] Mummaneni PV, Haid RW (2005). Atlantoaxial fixation: overview of all techniques. Neurol India.

[20] Yeom JS, Buchowski JM, Kim HJ, Chang BS, Lee CK, Riew KD (2013). Risk of vertebral artery injury: comparison between C1-C2 transarticular and C2 pedicle screws. Spine J.

[21] Meyer D, Meyer F, Kretschmer T, Börm W (2012). Translaminar screws of the axis - an alternative technique for rigid screw fixation in upper cervical spine instability. Neurosurg Rev.

[22] Magerl F, Seemann P-S (1987). Stable Posterior Fusion of the Atlas and Axis by Transarticular Screw Fixation. Cervical Spine I.

[23] Paramore CG, Dickman CA, Sonntag VK (1996). The anatomical suitability of the C1-2 complex for transarticular screw fixation. J Neurosurg.

[24] Goel A, Laheri V (1994). Plate and screw fixation for atlanto-axial subluxation. Acta Neurochir (Wien).

[25] Khanfour AA, El Sekily NM (2015). Relation of the vertebral artery segment from C1 to C2 vertebrae: an anatomical study. Alexandria J Med.

[26] Ulm AJ, Quiroga M, Russo A (2010). Normal anatomical variations of the V₃ segment of the vertebral artery: surgical implications. J Neurosurg Spine [Internet.

[27] el-Sayed Hassanein GH (2013). Injury risk of vertebral artery during screw placement through Atlas posterior arch. Basic Sci Med.

[28] Mukesh S, Prabhat G, Mohd Salahuddin A, Kumar SR (2014). Distance between midline and vertebral artery groove of atlas - a real aid to the neurosurgeon. J Surg Academia.

[29] Gawad FAA El, Shaaban MH, Shuaib DM, Shallan HM (2019). Anatomical variations of the vertebral artery and its relation to the atlas vertebra: radiological and dry bone study. Eur J Anat.

